# Urban–Rural Differences in Clinical Characteristics of Prostate Cancer at Initial Diagnosis: A Single-Center Observational Study in Anhui Province, China

**DOI:** 10.3389/fonc.2021.704645

**Published:** 2021-08-03

**Authors:** Qi Long Song, Yinfeng Qian, Xuhong Min, Xiao Wang, Jing Wu, Xiaohu Li, Yongqiang Yu

**Affiliations:** ^1^Department of Radiology, The First Affiliated Hospital of Anhui Medical University, Hefei, China; ^2^Department of Radiation Oncology, Anhui Chest Hospital, Hefei, China

**Keywords:** place of residence, prostate cancer, risk factors, screening, early detection

## Abstract

**Background:**

People residing in rural areas have higher prostate cancer (PCa) mortality to incidence ratio (M/I) and worse prognosis than those in urban areas of China. Clinical characteristics at initial diagnosis are significantly associated with biochemical recurrence, overall survival, and PCa disease-free survival.

**Objective:**

This study aimed at investigating the clinical characteristics at initial diagnosis of urban and rural PCa patients and to establish a logistic regression model for identifying independent predictors for high-grade PCa.

**Materials and Methods:**

Clinical characteristics for PCa patients were collected from the largest prostate biopsy center in Anhui province, China, from December 2015 to March 2019. First, urban–rural disparities in clinical characteristics were evaluated at initial diagnosis. Second, based on pathological findings, we classified all participants into the benign+ low/intermediate-grade PCa or high-grade PCa groups. Univariate and multivariate logistic regression analyses were performed to identify independent factors for predicting high-grade PCa, while a nomogram for predicting high-grade PCa was generated based on all independent factors. The model was evaluated using area under receiver-operating characteristic (ROC) curve as well as calibration curve analyses and compared to a model without the place of residence factor of individuals.

**Results:**

Statistically significant differences were observed between urban and rural PCa patients with regard to tPSA, PSA density (PSAD), and Gleason score (GS) (p < 0.05). Logistic regression analysis revealed that tPSA [OR = 1.060, 95% confidence interval (CI): 1.024, 1.098], PSAD (OR = 14.678, 95%CI: 4.137, 52.071), place of residence of individuals (OR = 5.900, 95%CI: 1.068, 32.601), and prostate imaging reporting and data system version 2 (PI-RADS v2) (OR = 4.360, 95%CI: 1.953, 9.733) were independent predictive factors for high-grade PCa. The area under the curve (AUC) of the nomogram was greater than that of the model without the place of residence of individuals. The calibration curve of the nomogram indicated that the prediction curve was basically fitted to the standard curve, suggesting that the prediction model had a better calibration ability.

**Conclusions:**

Compared to urban PCa patients, rural PCa patients presented elevated tPSA, PSAD levels, and higher pathological grades. The place of residence of the individuals was an independent predictor for high-grade PCa in Anhui Province, China. Therefore, appropriate strategies, such as narrowing urban-rural gaps in access to health care and increasing awareness on the importance of early detection should be implemented to reduce PCa mortality rates.

## Introduction

Globally, there were about 1.3 million new prostate cancer (PCa) cases and 358,989 associated mortalities in 2018 (1). Compared to North America and Northern Europe, documented PCa incidences were much lower in China (2). This could be attributed to environmental differences and/or protective effects of diets. It may also be due to a lack of PCa screening and early detection. PCa mortality-incidence ratio (M/I), as a parameter for evaluating the prognosis of PCa, has significantly decreased substantially in North America, coinciding with the initiation of widespread prostate-specific antigen (PSA) screening (3, 4). However, PCa M/I has always been high in China. According to the World Health Organization, there were 603,000 new documented PCa cases in mainland China and approximately 266,000 mortalities in 2015 (5). The PCa M/I ratio in China was about 44.11%, compared to only 11.65% in America (6). This M/I difference may be attributed to substantive genetic differences; however, this does not fully explain for the huge urban–rural disparity in PCa M/I ratio in China. In China, rural men exhibited higher M/I ratios as a result of PCa compared to men in urban areas. It has been reported that PCa M/I ratio was approximately 60% in rural areas, nearly three times higher than that in urban areas (7). Establishing the causes of this big disparity may improve health care services and reduce PCa M/I. Survival outcomes for PCa are significantly correlated with various factors, such as serum PSA levels and Gleason scores (GS) (8, 9). In the new five-tiered prostate cancer grading system proposed by Epstein and colleagues, high-grade PCa (GS ≥ 8) has a much higher likelihood for biochemical recurrence (BCR) following radical prostatectomy (RP) and a much higher mortality rate (10, 11).

Studies, including the European Randomized Study of Screening for Prostate Cancer (ERSPC), Prostate Cancer Prevention Trial risk calculation (PCPT-RC), and nomograms, have developed various predictive models for prostate biopsy results (12–14). However, these models have various limitations when applied in China. First, substantive differences in PSA levels exist between Western and Asian populations. Second, MP-MRI multiparametric MRI (MP-MRI) is rarely included in these models. Third, considering PCa M/I ratio differences between urban and rural areas, it is necessary to include the place of residence of the individuals into high-grade PCa models. In this study, we used data from the largest prostate biopsy center in Anhui province located inland in the eastern midlands of China. We investigated how clinical data at initial diagnosis vary between urban and rural PCa patients and if place of residence is an independent predictor for high-grade PCa. Finally, using the identified independent factors, we established a predictive model for high-grade PCa.

## Materials and Methods

### Study Area and Population

**Figure 1:**
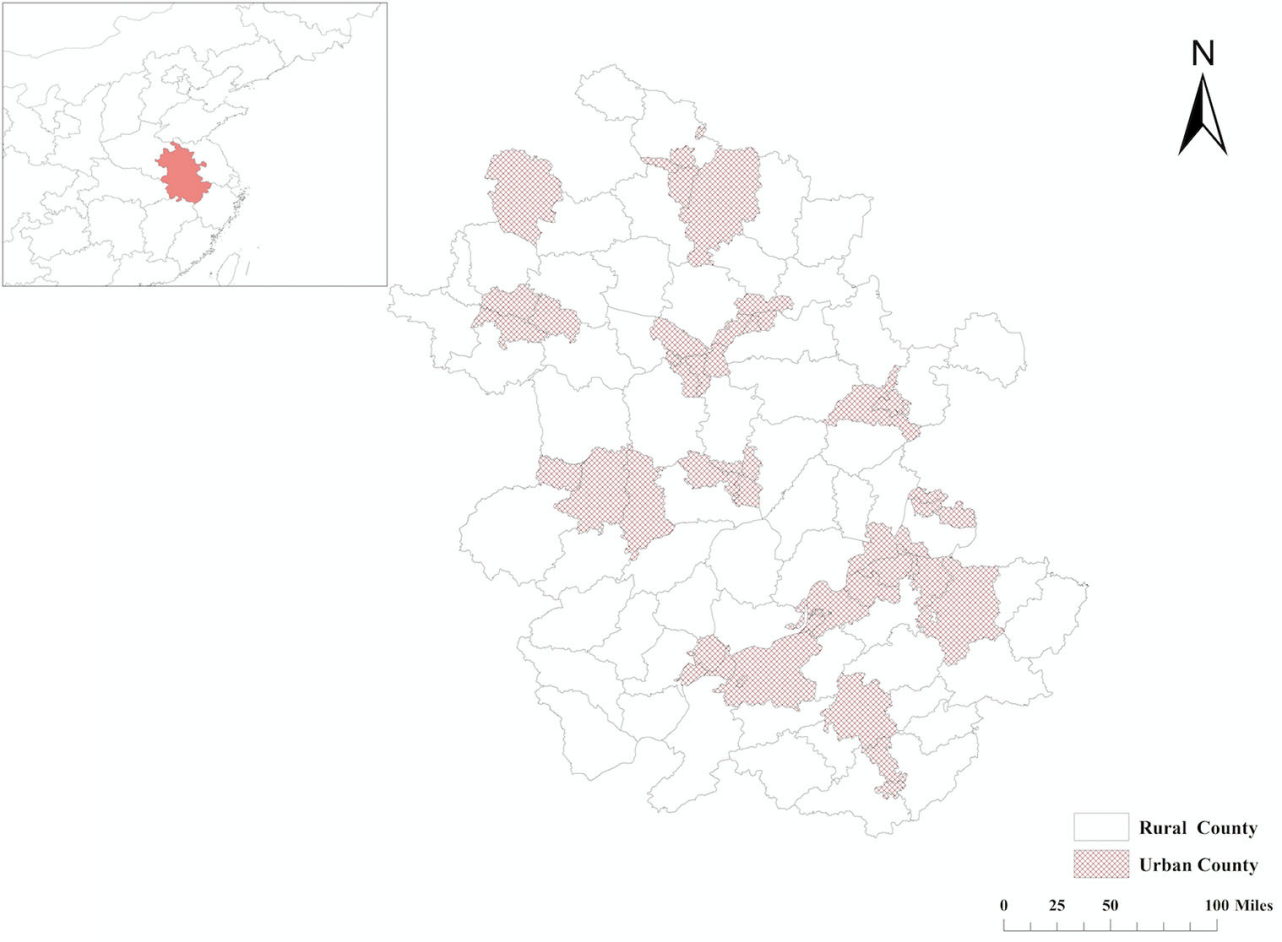
Distribution of urban and rural counties in Anhui Province, China.

**Figure 2:**
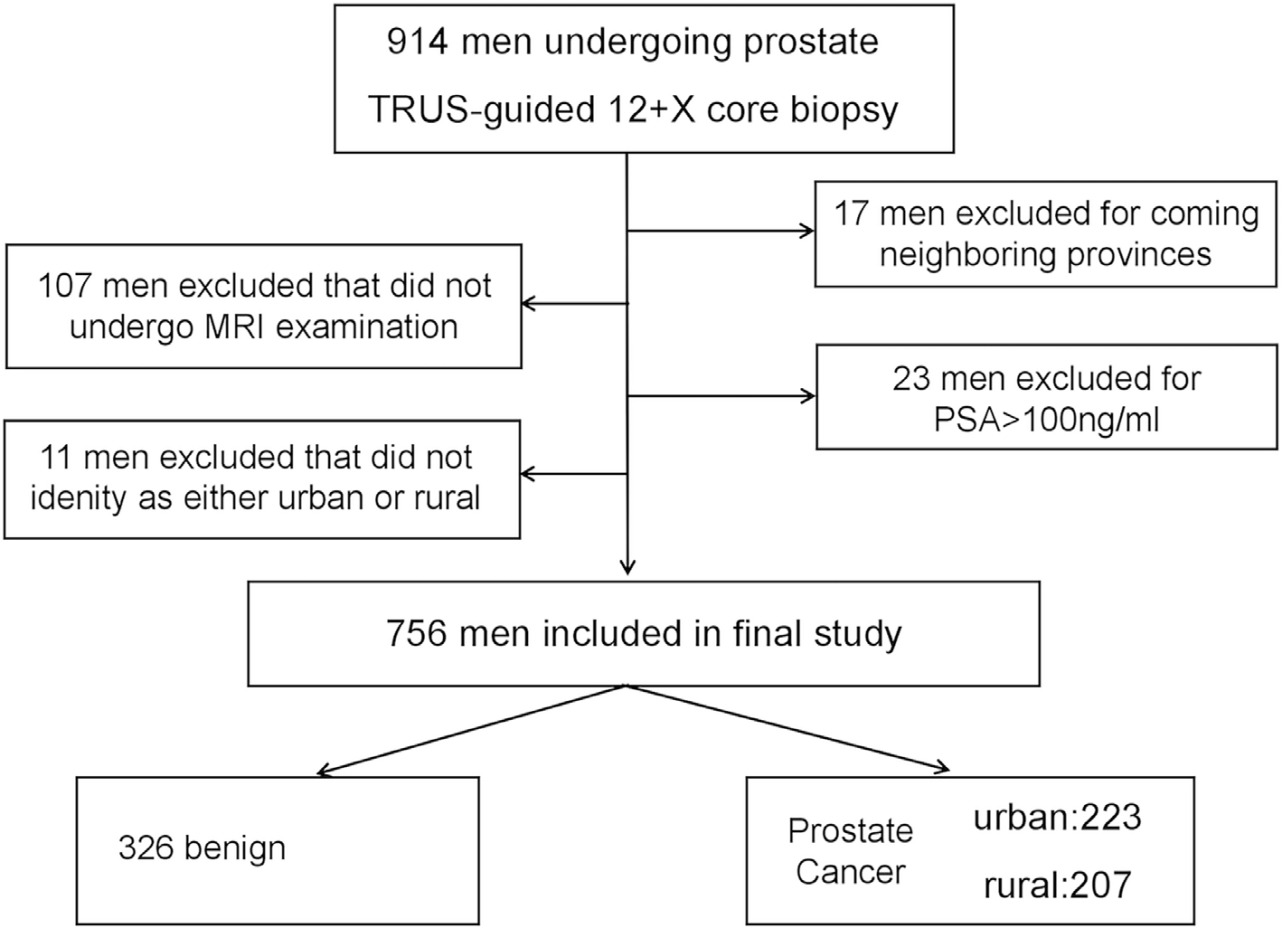
Flow chart schematic of study recruitment and retention.

Ethical approval for this study was obtained from the institutional review board of the First Affiliated Hospital of Anhui Medical University, China. The study was performed in Anhui province, which had a total area of 140,100 km^2^ and had a population of 63.7 million people in 2019. Based on the categorization of districts and counties of Anhui Planning Bureau, there were 104 counties in Anhui Province, which included 45 urban counties and 59 rural counties (**Figure 1**). This was a retrospective study involving 914 participants undergoing diagnostic prostate transrectal ultrasound (TRUS)-guided 12+*X* core biopsy at our institution. The study was performed from December 2015 to March 2019. Seventeen participants were excluded as they were from neighboring provinces. Participants with PSA >100 ng/dl were excluded as extreme values (*N* = 23). Participants without MP-MRI examination data were also excluded (*N* = 107). Eleven participants who did not identify as urban or rural residents were also excluded. Therefore, the final analysis involved a total of 756 participants from 91 counties, 38 of them urban and 53 rural. The flow chart for participant recruitment and retention process is shown in **Figure 2**.

### Collection of Clinical Characteristics

Data for age, tPSA, fPSA, (f/t)PSA, PSA density (PSAD), prostate imaging reporting and data system version 2 (PI-RADS v2), body mass index (BMI), left-right, anteroposterior, and vertical prostate diameter, GS, and place of residence of the individual were extracted from the electronic medical record systems of the hospital and Picture Archiving and Communication Systems (PACS). Prostate volume was measured using a 3.0-T MRI system (GE 3.0T HDxt) and calculated as follows: volume = left-right diameter × vertical diameter × anteroposterior diameter × π/6. MRI images were centrally reviewed by two experienced radiologists with 5 and 7 years of experience in interpreting prostate MRI, respectively, using PI-RADS v2. The radiologists were blinded to the clinical data of the participants. When PI-RADS v2 categories were inconsistent, an arbitration expert (more senior radiologist with >15 years of experience) made the final decision. Based on the level of suspicion or risk of clinically significant prostate cancer (CSPCa), MRI results were divided into five categories (15).

### Prostate Biopsy and Pathological Diagnosis

All participants were subjected to TRUS-guided 12-core systematic biopsy (SB). In case suspected malignant nodules were seen on ultrasound or MRI images, additional two to four needle biopsies were performed in regions with abnormal echoes or signals. The TRUS-guided biopsy was performed as described by Presti JR (16). Based on the latest International Society of Urological Pathology (ISUP) Gleason Grading Group (GGG), pathological diagnoses were classified as benign, ISUP 1 (GS 6), ISUP 2 (GS 3 + 4), ISUP 3 (GS 4 + 3), ISUP 4 (GS 8), and ISUP 5 (GS 9–10) (17). We defined ISUP >3 as high-grade PCa based on previous studies (11, 12).

### Statistical Analysis

Statistical analyses were performed using the IBM SPSS 23.0 software (IBM), and additional graphics was generated in R version 3.5.1 (http://www.r-project.org). Normality of continuous clinical data distribution was tested through the Shapiro–Wilk test. For continuous data, t-test and non-parametric Mann–Whitney U tests were performed. The Chi-Square test was used for categorical data. Ranked data were analyzed using the Mann–Whitney U test. Multivariate logistic regression analyses were performed using the stepwise forward strategy to filter independent factors for high-grade PCa based on significant factors as determined by univariate analysis. Finally, a nomogram was developed using the Cox proportional hazards model and validated using area under the receiver-operating characteristic (ROC) curve and calibration curve analyses. The model was compared to the model lacking place of residence as a factor. *P* < 0.05 was set as the threshold for statistical significance.

## Results

### Clinical Characteristics of the Study Population

A total of 756 participants with valid clinical data on age, tPSA, fPSA, PSAD, (f/t) PSA, prostate volume, BMI, PI-RADS v2, GS, and place of residence were enrolled in this study. Mean age, tPSA, fPSA, (f/tPSA), PSAD, prostate volume, and BMI were 70.46 ± 8.07 years old, 23.73 ± 11.78 ng/ml, 5.32 ± 3.13 ng/ml, 0.24 ± 0.16, 0.50 ± 0.29 ng/ml^2^, 51.26 ± 28.50 ml, and 23.10 ± 3.07, respectively. Among the participants, 44.2% were rural residents. Proportions of PI-RADS v2 categories 1–2, 3, 4, and 5 were 20.2, 31.4, 27.9, and 20.5, respectively. The proportions of benign, ISUP 1, ISUP 2, ISUP 3, ISUP 4, and ISUP 5 were 43.1, 5.8, 11.4, 12.7, 14.4, and 12.6%, respectively. Clinical characteristics at initial diagnosis were as shown in **Tables 1** and **2**.

**Table 1:** Clinical characteristics of study participants.

Characteristic	Overall					PCa			
	Total (n = 756)	Benign+low/intermediate-grade PCa (n = 552)	High-grade PCa (n = 204)	t/χ^2^	*P*-value	Urban (n = 223)	Rural (n = 207)	t/χ^2^	*P*-value
Age, years	70.46 ± 8.07	69.93 ± 7.57	71.88 ± 9.14	−2.722^a^	0.007	71.34 ± 9.02	70.45 ± 8.43	1.055^a^	0.292
tPSA, ng/ml	23.73 ± 11.78	21.73 ± 9.87	29.14 ± 14.52	−6.736^a^	<0.001	24.02 ± 10.23	28.42 ± 13.19	−3.882^a^	<0.001
fPSA, ng/ml	5.32 ± 3.13	4.93 ± 2.68	6.39 ± 3.97	−4.859^a^	<0.001	5.56 ± 3.46	6.07 ± 3.84	−1.449^a^	0.148
(f/t)PSA	0.24 ± 0.16	0.24 ± 0.16	0.23 ± 0.17	0.750^a^	0.454	0.24 ± 0.17	0.22 ± 0.15	1.296^a^	0.196
PSAD, ng/ml^2^	0.50 ± 0.29	0.43 ± 0.21	0.69 ± 0.38	−9.264^a^	<0.001	0.55 ± 0.31	0.64 ± 0.35	−2.797^a^	0.005
Volume, ml	51.26 ± 28.50	52.77 ± 29.12	47.16 ± 26.33	2.525^a^	0.012	50.74 ± 28.16	48.89 ± 27.87	0.684^a^	0.494
BMI	23.10 ± 3.07	23.19 ± 3.02	22.87 ± 3.21	1.271^a^	0.204	23.05 ± 3.14	22.91 ± 3.08	0.466^a^	0.641
Rural residence	334 (44.2%)	196 (35.5%)	138 (67.6%)	62.391^b^	<0.001	0 (0.0%)	207 (100.0%)	430.000^b^	<0.001

PSAD, PSA density; ^a^: t value; ^b^: χ2 value.

**Table 2:** PI-RADS v2 and biopsy results of study participants.

Characteristic	Overall					PCa			
	Total (n = 756)	Benign+low/intermediate-grade PCa (n = 552)	High-grade PCa (n = 204)	Z	*P*-value	Urban (n = 223)	Rural (n = 207)	Z	*P*-value
PI-RADS v2				−12.955	<0.001			−1.274	0.203
1–2	153 (20.2%)	151 (27.4%)	2 (1.0%)			7 (3.1%)	3 (1.4%)		
3	237 (31.4%)	204 (40.0%)	33 (16.2%)			49 (22.0%)	41 (19.8%)		
4	211 (27.9%)	138 (25.0%)	73 (35.8%)			87 (39.0%)	78 (37.7%)		
5	155 (20.5%)	59 (10.7%)	96 (47.1%)			80 (35.9%)	85 (41.1%)		
Biopsy results				−22.131	<0.001			−8.192	<0.001
Benign	326 (43.1%)	326 (59.1)							
ISUP 1	44 (5.8%)	44 (8.0%)				36 (16.1%)	8 (3.9%)		
ISUP 2	86 (11.4%)	86 (15.6%)				62 (27.8%)	24 (11.6%)		
ISUP 3	96 (12.7%)	96 (17.4%)				59 (26.5%)	37 (17.9%)		
ISUP 4	109 (14.4%)		109 (53.4%)			42 (18.8%)	67 (32.4%)		
ISUP 5	95 (12.6%)		95 (46.6%)			24 (10.8%)	71 (34.3%)		

ISUP, International Society of Urological Pathology.

### Clinical Characteristics of Urban and Rural PCa Subgroups

Of the 430 participants diagnosed with PCa, 223 were urban residents while 207 were rural residents. Mean tPSA and PSAD of rural patients were higher than those of their urban counterparts (*p* < 0.001, *p* = 0.005). Additionally, rural patients exhibited a higher pathologic grade than urban patients (*p* < 0.001). However, there were no significant differences between urban and rural subgroups with regard to age (*p* = 0.292), fPSA (*p* = 0.148), (f/t)PSA (*p* = 0.196), volume (*p* = 0.494), BMI (*p* = 0.641), and PI-RADS v2 (*p* = 0.203) (**Tables 1** and **2**).

### Models for Predicting High-Grade PCa

**Figure 3:**
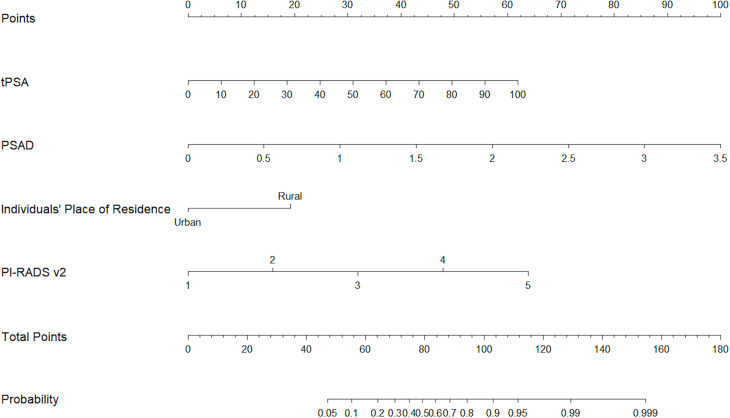
The nomogram for predicting prostate biopsy results for high-grade PCa.

**Figure 4:**
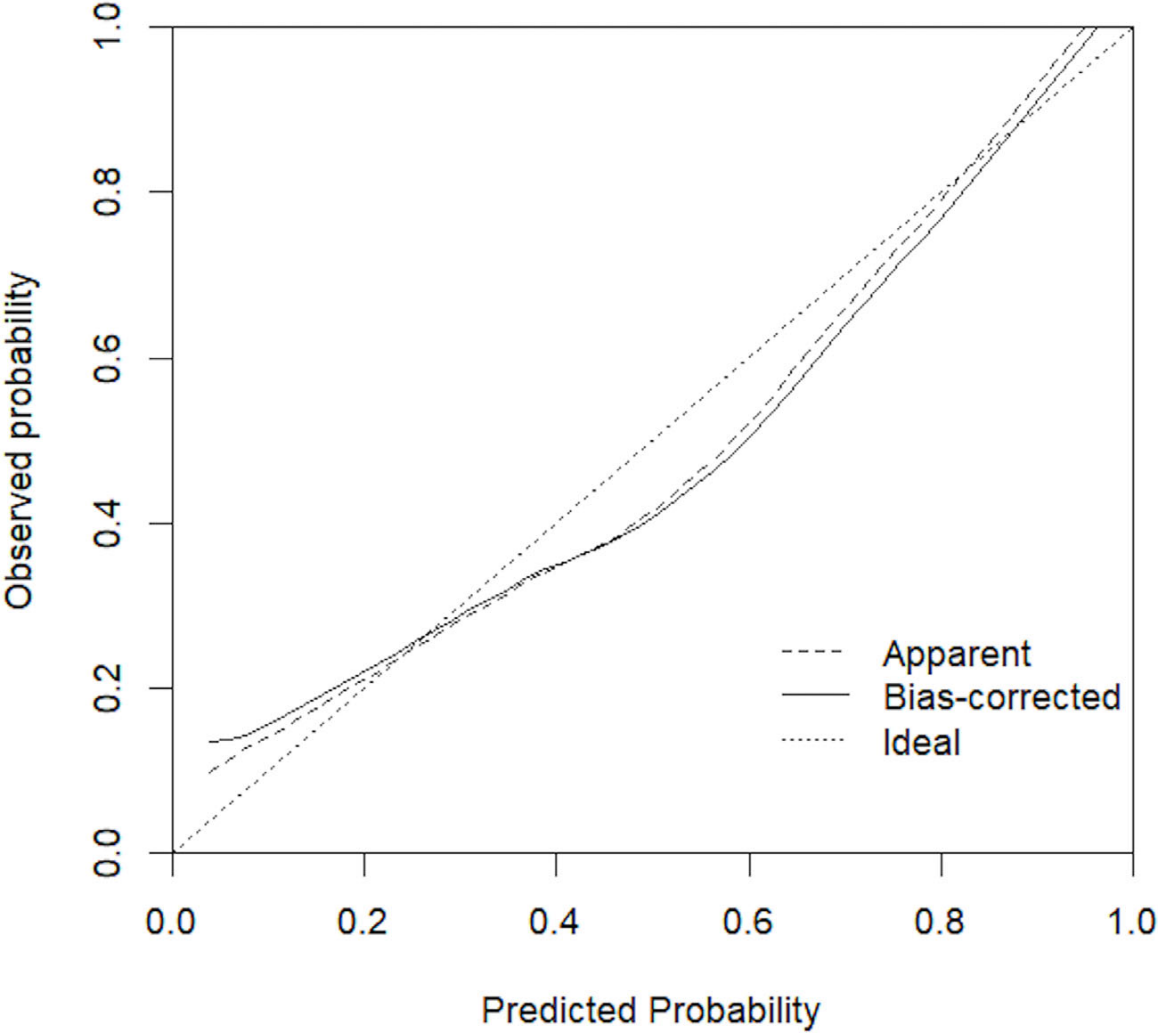
Calibration curve for the nomogram model.

**Figure 5:**
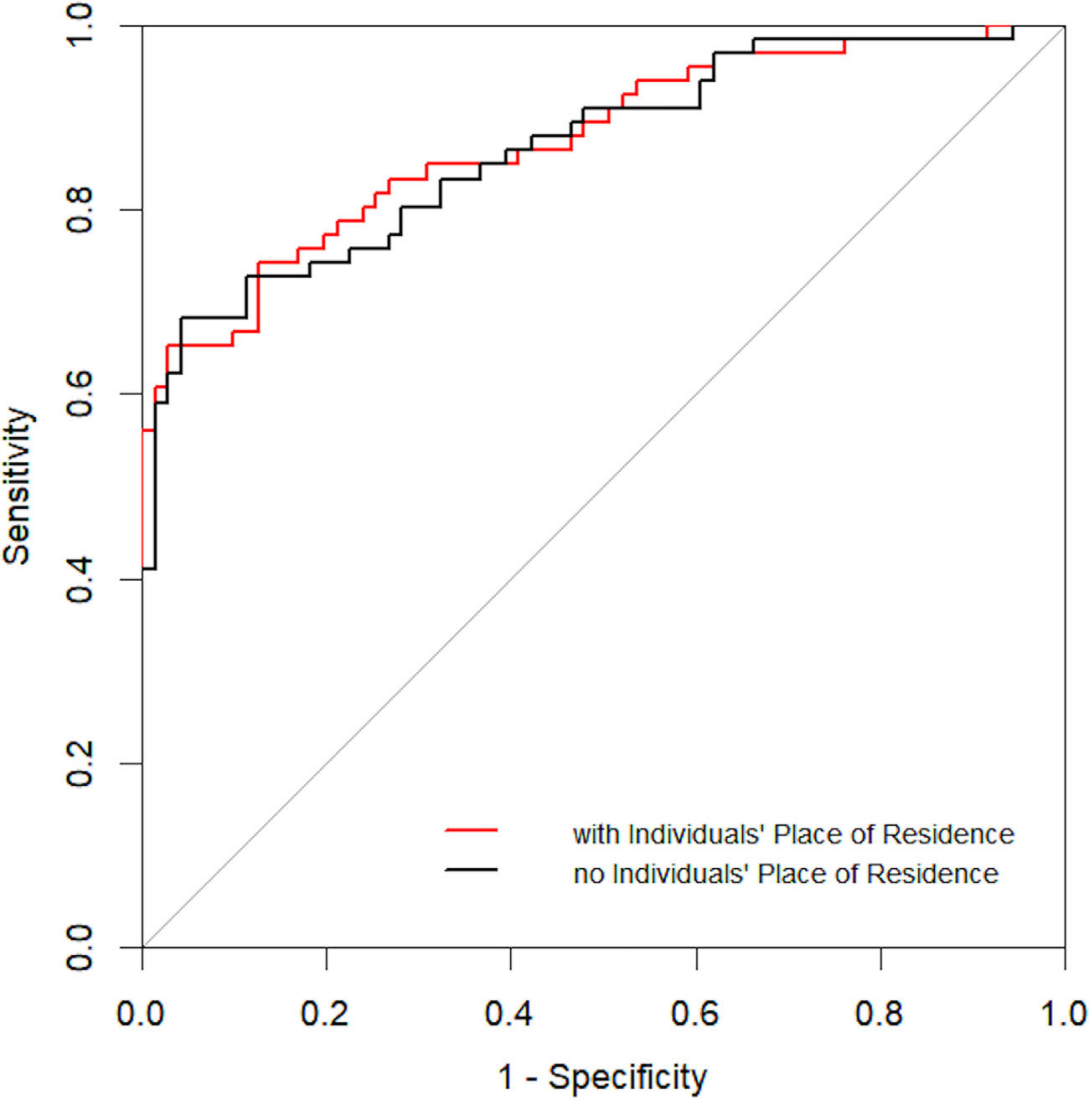
ROC curve for independent risk factors with and without place of residence of individuals.

Based on pathologic findings, participants were grouped into benign + low/intermediate-grade PCa group (n = 552) or high-grade PCa group (n = 204). In the univariate analysis, age, tPSA, fPSA, PSAD, volume, place of residence of the individual, and PI-RADS v2 reached the pre-specified criteria (*p* < 0.05) for use in stepwise multivariate analysis. Multivariate analysis identified tPSA, PSAD, place of residence, and PI-RADS v2 as independent predictors for high-grade PCa (**Table 3**). Next, tPSA, PSAD, and PI-RADS v2 were used to establish model 1 for predicting high-grade PCa. Then, places of residence of individuals were added to predictors in model 1 to generate model 2. **Figure 3** shows the nomogram based on independent predictors in model 2. The nomogram had a c-index of 0.874. The criteria for using the nomogram: A point corresponded to each independent predictor while total points corresponded to the predicted risk value. For example: for an urban residence, if tPSA was 10 ng/ml, while PSAD was 0.25 ng/ml^2^, PI-RADS v2 category was 2, the corresponding points were 0, 12, 12, 30, then, total points were 54; the corresponding prostate biopsy result was about 10% of high-grade PCa. Calibration curve of the nomogram revealed that the prediction curve was basically fitted to the standard curve, suggesting that the prediction model had better calibration ability (**Figure 4** **).** The area under the ROC curve for model 1 was 0.867 (95% CI: 0.806, 0.927, *p* < 0.001) and 0.874 (95% CI: 0.815, 0.933, *p* < 0.001) for model 2. The model with places of residence exhibited slightly better predictive values than the one without (**Figure 5**).

**Table 3:** Results of logistic regression analysis.

Characteristics	B	OR (95% CI)	*P*-value
Age	0.024	1.024 (0.973, 1.078)	0.360
tPSA	0.058	1.060 (1.024, 1.098)	0.001
fPSA	0.018	1.018 (0.908, 1.142)	0.758
PSAD	2.686	14.678 (4.137, 52.071)	<0.001
Volume	−0.005	0.995 (0.979, 1.010)	0.503
Place of residence	1.775	5.900 (1.068, 32.601)	0.031
PI-RADS v2	1.472	4.360 (1.953, 9.733)	<0.001

PSAD, PSA density; OR, odds ratio.

## Discussion

To the best of our knowledge, this is the first study to show urban–rural disparities in PCa clinical characteristics at initial diagnosis worldwide. An earlier study indicated that men in rural areas had significantly higher PCa mortality than their urban-resident counterparts (7). A previous study in Shanghai found that the 5-year observed survival (OS) and relative survival (RS) for PCa were higher in urban areas relative to rural areas (18). The reasons for these outcomes have not been established; however, they could be due to the nature of the underlying cancer registry data that focus on morbidity and mortality and not on clinical characteristics at initial diagnosis. Our findings suggest this urban–rural disparity in PCa M/I may be due to differences in clinical characteristics at initial diagnosis.

Studies have evaluated various risk factors for PCa and high-grade PCa (19–21); however, based on the high PCa M/I in China and the huge PCa M/I ratio disparity between urban and rural areas, it is important to consider the impact of place of residence on high-grade PCa. We found that tPSA, PSAD, PI-RADS v2, and place of residence were independent predictors for high-grade PCa, consistent with a previous study in Jiangsu Province, China, showing that PSA levels and tumor grade at diagnosis were significantly independently correlated with PCa disease-specific survival (9). Our findings support the concept that more emphasis should be directed toward the differences that exist between urban and rural PCa patients or underlying healthcare disparities, especially late PCa screening and detection, faced by rural men. Since it is associated with over-diagnosis and over-treatment in North America and Northern Europe, it has not been established whether PSA is beneficial in clinical diagnosis and treatment (22–24). However, in China, PCa screening is insufficient, especially in rural areas. Moreover, poor PSA screening results in a significant proportion of PCa patients do not offer the best opportunity for treatment, resulting in higher M/I ratio.

In this study, PI-RADS v2 did not differ significantly in urban *vs*. rural PCa patients, although it had a high sensitivity and specificity for CSPCa when the cutoff value was category 4 (25, 26). This may be because GS 6 lesions were abundant in all PI-RADS v2 categories and GS ≥7 were more often seen in PI-RADSv2 ≥4 (27). In this study, PI-RADS v2 categories of PCa in both urban and rural cases were found to be concentrated at PI-RADS v2 ≥4 (urban: 167/223; rural: 163/207). Additionally, the AUC for model 2 was slightly higher than that of model 1 probably because PI-RADS v2 and PSAD also had higher odds ratios (ORs) for high-grade PCa. However, we cannot deny that place of residence significantly influences PCa outcomes in China.

Behind the clinical characteristics at initial diagnosis are socioeconomic factors related to urbanization, including per capita disposable income (PCDI), medical insurance reimbursement level, health care services, and health literacy. A study evaluating health provider–patient communication for PCa screening found that financial status was significantly associated with PCa screening and early detection (28). There were socioeconomic and health insurance disparities between urban and rural areas of China. The annual PCDI of urban residents was found to be nearly three times higher than that of rural residents in 2015, at $5031 and $1842, respectively (29). In China, social health insurance dominates the health insurance market. There are three types of social insurance programs (30): the Urban Employee Basic Medical Insurance (UEBMI) launched in 1998, which covers urban employees and retired people; the Urban Resident Basic Medical Insurance (URBMI) established in 2007, which covers unemployed urban residents, and the New Rural Cooperative Medical Scheme (NRCMS) initiated in 2003, which covers local rural residents. Rural residents have fewer comprehensive benefits than urban residents, and the reimbursement level for hospitalized patients covered by the NRCMS (48.04%) is significantly lower than that of UEBMI (74.64%) and URBMI (59.23%) (31). The lower reimbursement ratios limit the ability for rural patients to obtain PCa diagnosis before the appearance of clinical symptoms, such as lower urinary tract obstruction, pain during urination, or bone pain. An uneven distribution of healthcare services plays an important role in disparities in early PCa detection between urban and rural areas of China. Rural men account for a large portion of high-grade PCa but are exposed to fewer medical resources in terms of medical staff and institutions. According to the China Health Statistics Yearbook 2015, the number of registered doctors per thousand people in urban areas is 2.57 times higher than in rural areas (32). The number of hospitals in urban areas increased by 66% from 2005 to 2017, whereas the number of health clinics in rural areas only increased by 8% in the same period (33). Furthermore, tertiary hospitals that can offer prostate biopsy are mainly localized in urban areas. The longer travel distance may deter rural men from accessing early PCa detection services. Education also accounts for disparities in urban–rural health status. Chinese rural residents have relatively poor health literacy levels. Health literacy is associated with health beliefs, including attitudes, values, and knowledge that individuals possess on screening and early detection, which influence their perception of the need for early diagnosis.

This study has several limitations. First, although we discussed several possible explanations for urban–rural disparities in clinical characteristics at initial diagnosis, it is possible that other factors, such as occupation and environmental factors, are also involved. Second, this is a single center study in China, albeit a relatively large one, and multicenter data can externally validate our findings. Additionally, this is a retrospective study on urban–rural disparities in PCa clinical characteristics at initial diagnosis; other factors may also affect the prognosis of patients, and these will be addressed in future studies.

Based on clinical characteristics at initial diagnosis, we investigated disparities in urban–rural PCa M/I ratios. We found that rural PCa patients presented higher tPSA, higher PSAD, and higher pathological grades when compared to urban patients and that rural residence was an independent risk factor for high-grade PCa in Anhui Province, China. Our findings involve the development of appropriate strategies to reduce PCa mortality, such as narrowing urban–rural gaps in accessing healthcare and increasing awareness on the importance of early detection.

## Data Availability Statement

The original contributions presented in the study are included in the article/supplementary material. Further inquiries can be directed to the corresponding authors.

## Author Contributions

YY and XL designed the study, and helped to drafted the manuscript. QS, YQ, JW, and XW were involved in data collection and assembly. YY and XM performed data analysis and interpretation. QS drafted the manuscript. All authors contributed to the article and approved the submitted version.

## Funding

This study was financially supported by Central Government Transfer Payment Financial Construction Project (Z155080000004) and the National Natural Science Foundation of China (81771817).

## Conflict of Interest

The authors declare that the research was conducted in the absence of any commercial or financial relationships that could be construed as a potential conflict of interest.

## Publisher’s Note

All claims expressed in this article are solely those of the authors and do not necessarily represent those of their affiliated organizations, or those of the publisher, the editors and the reviewers. Any product that may be evaluated in this article, or claim that may be made by its manufacturer, is not guaranteed or endorsed by the publisher.
